# Conductive Polymer Synthesis with Single-Crystallinity via a Novel Plasma Polymerization Technique for Gas Sensor Applications

**DOI:** 10.3390/ma9100812

**Published:** 2016-09-30

**Authors:** Choon-Sang Park, Dong Ha Kim, Bhum Jae Shin, Do Yeob Kim, Hyung-Kun Lee, Heung-Sik Tae

**Affiliations:** 1 School of Electronics Engineering, College of IT Engineering, Kyungpook National University, Daegu 702-701, Korea; purplepcs@ee.knu.ac.kr (C.-S.P.); ao9o9@ee.knu.ac.kr (D.H.K.); 2 Department of Electronics Engineering, Sejong University, Seoul 143-747, Korea; hahusbj@sejong.ac.kr; 3 Nano Convergence Devices Research Department, Electronics and Telecommunications Research Institute (ETRI), Daejeon 34129, Korea; nanodykim@etri.re.kr (D.Y.K.); hklee@etri.re.kr (H.-K.L.)

**Keywords:** atmospheric pressure plasma, plasma-polymerized pyrrole, single-crystalline, gas sensor, iodine doping

## Abstract

This study proposes a new nanostructured conductive polymer synthesis method that can grow the single-crystalline high-density plasma-polymerized nanoparticle structures by enhancing the sufficient nucleation and fragmentation of the pyrrole monomer using a novel atmospheric pressure plasma jet (APPJ) technique. Transmission electron microscopy (TEM), Fourier transform infrared spectroscopy (FT-IR), X-ray photoelectron spectroscopy (XPS), and field emission scanning electron microscopy (FE-SEM) results show that the plasma-polymerized pyrrole (pPPy) nanoparticles have a fast deposition rate of 0.93 µm·min^−1^ under a room-temperature process and have single-crystalline characteristics with porous properties. In addition, the single-crystalline high-density pPPy nanoparticle structures were successfully synthesized on the glass, plastic, and interdigitated gas sensor electrode substrates using a novel plasma polymerization technique at room temperature. To check the suitability of the active layer for the fabrication of electrochemical toxic gas sensors, the resistance variations of the pPPy nanoparticles grown on the interdigitated gas sensor electrodes were examined by doping with iodine. As a result, the proposed APPJ device could obtain the high-density and ultra-fast single-crystalline pPPy thin films for various gas sensor applications. This work will contribute to the design of highly sensitive gas sensors adopting the novel plasma-polymerized conductive polymer as new active layer.

## 1. Introduction

Nanostructured conductive polymers, such as polyaniline (PANI), polypyrrole (PPy), polythiophene (PTh), and poly(3,4-ethylenedioxythiophene) (PEDOT), have been studied as active layers of gas sensors [1,2,3,4,5,6,7,8,9,10,11,12]. Conductive polymers can be synthesized through the chemical method, electrochemical synthesis, electrospinning, seeding polymerization, interfacial polymerization, and low pressure plasma polymerization techniques [13,14,15,16]. However, almost all polymerization techniques have high synthesis temperatures (100–600 °C) with solutions or the wet process [16]. Therefore, conventional conducting polymers have a generally amorphous state or poly-crystalline characteristics with non-porous properties due to high temperature, and present difficulties in their incorporation in highly sensitive gas sensors. However, nano-porous polymers can be synthesized by atmospheric pressure plasma jets (APPJs) due to the process of removing thermal solutions [17,18,19,20,21,22].

However, it is difficult to synthesize polymers using conventional APPJs because the plasma produced in ambient air conditions has low energy [23,24,25,26]. As a result, they would not feed enough energy for the nucleation of the monomers. Accordingly, most polymer films synthesized by the conventional APPJs tend to show poor film qualities, such as a low molecular weight and weak chemical stabilities with an amorphous state. More importantly, conductive polymer nanoparticles and nanofibers synthesized by the APP technique have non-crystalline or poly-crystalline characteristics [27,28,29,30]. To the authors’ knowledge, there have been no previous reports on the synthesis of porous conductive polymer nanoparticles with single-crystalline characteristics using APP polymerization techniques at room temperature due to insufficient plasma energy for the nucleation of monomers. Therefore, it is necessary to enhance the plasma energy to synthesize high-density conductive polymer nanoparticles with single-crystallinity as the active layers for gas sensors on various substrates at room temperature.

We have recently reported a new polymer synthesis method using APPJs with an additional plastic tube and bottom cap [31]. The plasma-polymerized aniline (pPANI) nanofibers and nanoparticles were reported to be successfully obtained with high molecular weights and poly-crystalline characteristics [31]. However, previous APPJs with plastic tubes were not sufficiently completed for the synthesis of single-crystalline characteristics. In this study, we introduce an initial synthesis of high-density pyrrole (pPPy) nanoparticles with single-crystallinity on various substrates, such as glass, plastic, and interdigitated gas sensor electrodes, using a novel atmospheric pressure plasma polymerization technique at room temperature using an additional glass tube with a higher dielectric constant to increase the charged particles by the plasmas. In addition, the synthesized pPPy was doped with iodine to introduce charge carriers into the plasma-polymerized structures to render them conductive [32,33,34]. In particular, we examined the conductivity variation of the iodine-doped pPPy grown on the interdigitated gas sensor electrodes in order to check the suitability of the active layer for the electrochemical toxic gas sensors. Our experimental results show that the single-crystalline high-density pPPy nanoparticles can be obtained using the novel APPJ device with a single bundle of three glass tubes to enhance the plasma jets in the nucleation region.

## 2. Experimental Section

### 2.1. Plasma Polymer Synthesis and Measurement

Argon gas (99.999%) was used as the plasma discharge gas at a flow rate of 1300 standard cubic centimeters per minute (sccm). Liquid pyrrole monomer (*M_w_* = 67 g·mol^−1^, Sigma-Aldrich Co., St. Louis, MO, USA) was vaporized using a glass bubbler, which was supplied by argon gas with a flow rate of 130 sccm. The three glass jets were tied and wrapped by copper tape electrode at 10 mm from the end of jet. The same sinusoidal power was applied to the powered electrode with a peak value of 12 kV with a frequency of 30 kHz on both outside and inside cases [31]. In both outside and inside cases, each glass jet had an inner diameter of 1.5 mm and an outer diameter of 3 mm. The center-to-center distance between each jet was 3 mm. The additional glass tube (or glass tube) had an inner diameter of 20 mm and a length of 60 mm. The polytetrafluoroethylene (PTFE) insulating substrate holder with an outer diameter of 15 mm was located outside or inside. The optical emission spectrometer (OES) was used to analyze the optical intensity and spectra of reactive nitrogen and oxygen peaks, respectively, for estimating the variations in the plasma energy states [35,36]. The surface temperature of the substrates was measured with an infrared thermometer (568 IR Thermometer, Fluke, Everett, WA, USA) using a special glass tube with a small hole. The power and monomer feeding system except for the additional glass tube employed in the novel APPJs in this research has been described in detail in [31].

### 2.2. Iodine Doping on Plasma Polymer Films

The plasma-polymerized pyrrole (pPPy) nanoparticle thin films on the substrates of interdigitated gas sensor electrodes were doped by iodine to introduce charge carriers into the plasma polymerized structures to render them conductive. The pPPy films were doped by placing samples in a sealed container containing solid iodine (1 g) under various iodine exposure (doping) times.

### 2.3. Scanning Electron Microscopy

The top and cross-section views images of pPPy nanoparticle thin films were measured by scanning electron microscopy (SEM, Hitachi SU8220, Tokyo, Japan) with accelerating voltage and current of 5 kV and 10 mA, respectively. The samples for SEM were imaged with a conductive platinum coating, which played a role in preventing the substrate from charging during the imaging process.

### 2.4. Trasmission Electron Microscopy

The high-resolution transmission electron microscopy (HRTEM) images and selected area electron diffraction (SAED) patterns were taken with a Titan G2 ChemiSTEM Cs Probe (FEI Company, Hillsboro, OR, USA) transmission electron microscope, operating at 200 kV. A TEM sample of pPPy nanoparticles was prepared by depositing a 6-µL solution (ultrasonically dispersed in DI water) on carbon-coated copper grids, and dried in air.

### 2.5. Fourier Transform Infrared Spectroscopy

The Fourier transform infrared spectroscopy (FT-IR) was used to determine the chemical changes introduced by the plasma. The FTIR were taken with a Perkin–Elmer Frontier spectrometer (PerkinElmer, Waltham, MA, USA) between 650 and 4000 cm^−1^.

### 2.6. X-ray Photoelectron Spectroscopy

The X-ray photoelectron spectroscopy (XPS) was carried out on a K-ALPHA surface analysis system (Thermo Fisher Scientific, Waltham, MA, USA), using a monochromatic Al Kα X-ray source (hυ = 1486.71 eV) operated at 15 kV and 20 mA. The pressure in the analyzing chamber was maintained at 10^−7^ Pa or lower during analysis, and the size of the analyzed area was 5 mm × 5 mm. Spectra were acquired with the angle between the direction of the emitted photoelectrons and the surface equal to 60°. The estimated analyzing depth of the used XPS set up was 8 to 10 nm. The high-resolution spectra were taken in the constant analyzer energy mode with a 200 eV for survey scan and a 50 eV pass energy for element scan, respectively. The value of 285.8 eV of the C1s core level was used for calibration of the energy scale.

## 3. Results and Discussion

**Figure 1:**
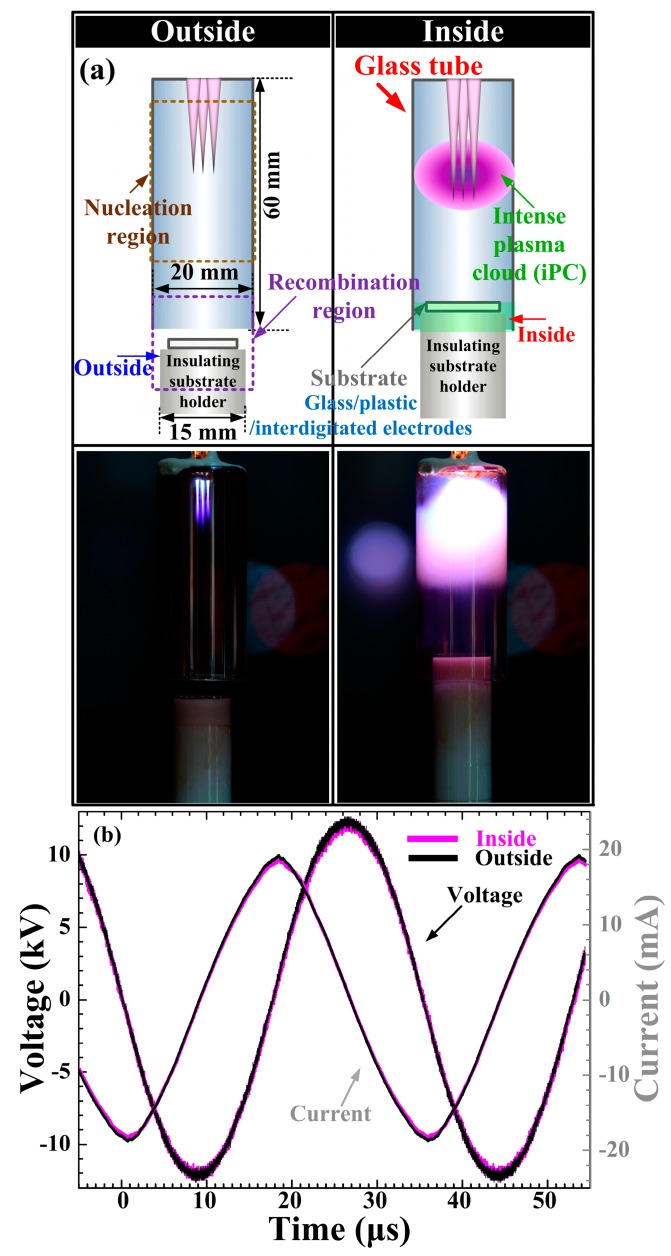
(**a**) Schematic diagram of experimental setup in this study and images of plasmas produced in the nucleation region; and (**b**) applied voltages and total currents of novel atmospheric pressure plasma jets (APPJs) whose insulating substrate holders (or polytetrafluoroethylene (PTFE) bottom cap) are placed outside or inside the glass tube.

As shown in Figure 1a, the newly proposed glass tube and cylindrical insulating substrate holder were introduced to minimize the quenching from ambient air and increase the plasma energy in the nucleation region. It is noted that the proposed glass tube and insulating substrate holder were installed at the jet end to confine the jet flow in the nucleation region and produce an intense and broad plasma. Moreover, it is well advised to leave a gap between the end of the glass tube and the substrate with the holder for a smoother jet flow. As shown in the plasma images (inside case), the profile of the produced plasma was dramatically changed, i.e., the strong, intense, and broad plasma was produced by adopting a jet with a glass tube, with a higher dielectric constant, instead of a plastic tube with a low dielectric constant [31]. Accordingly, thanks to an additional glass tube with a higher dielectric constant, the proposed APPJs can produce more efficient and intensive plasma in the nucleation region during plasma polymerization. As shown in Figure 1a, in the case of a jet whose insulating substrate holder is placed outside the glass tube (outside case), the streamer-like short plasmas were only produced in the nucleation region. On the contrary, the strong and intense plasma plumes were produced broadly and extended farther downstream in the case of a jet whose insulating substrate holder was placed about 5 mm inside the glass tube (inside case) even when the applied voltages and total currents (i.e., input power) were the same, as shown in Figure 1b.

**Figure 2:**
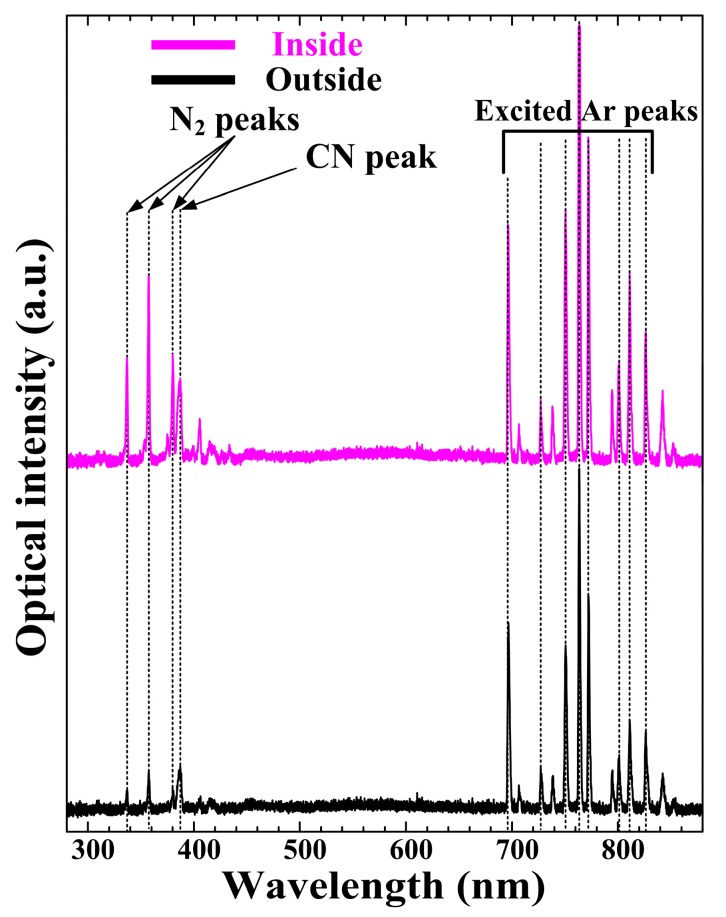
Optical emission spectra using optical emission spectrometer (OES) measured in the nucleation region of novel APPJs, whose insulating substrate holders are placed outside or inside the glass tube.

Figure 2 shows the optical emission spectra from 300 to 880 nm, measured in the nucleation region, further indicating that the excited N_2_, Ar, and carbonaceous species exist in the plasma plumes. Interestingly, the excited nitrogen second positive (N_2_; 337, 357, and 380 nm) peaks and the carbonaceous (CN; 388 nm) peak were significantly increased in the inside case. The various N_2_ peaks indicate a higher concentration of reactive nitrogen species (RNS), which have been shown to play an important role in obtaining a high-quality polymer layer. In addition, spectra from the carbonaceous species, such as CN (388 nm *B*^2^*Σ → X*^2^*Σ*), emitted during the nucleation processes of the pyrrole monomer, were significantly increased in the inside case. These OES data show that the proposed APPJs can be suitable for sufficient nucleation and fragmentation of the pyrrole monomer and the efficient generation of a new polymer without any high-temperature process or thermal damages.

**Figure 3:**
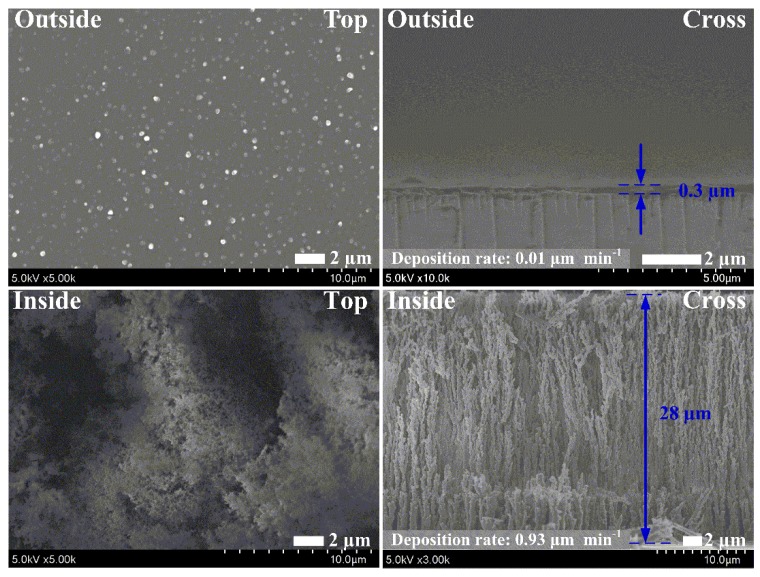
Changes in top and cross-section views of scanning electron microscopy (SEM) images of plasma-polymerized pyrrole (pPPy) nanoparticle thin films prepared via proposed APPJs after a deposition of 30 min in case of an adopting jet whose insulating substrate holders are placed outside or inside the glass tube. Scale bar = 2 µm.

Figure 3 shows the top-view and cross-section view SEM images of the pPPy nanofibers with a nanoparticle thin film, grown for 30 min, on plastic (for top-view) and glass (for cross-section view) substrates. The surface temperature of the substrates during the plasma deposition process in the inside case was about 30 °C. As shown in Figure 3, no nanoparticles or amorphous state was observed in the outside case because the weak plasma was produced in the nucleation region. However, in the inside case, many nanofibers and nanoparticles with porous states were observed to be linked together in uniform and upright networks, meaning that the proposed APPJs can efficiently synthesize the uniform nanofibers and nanoparticles. Generally, plasma polymer structures have many irregular cross-linked networks and porous networks. However, as shown in Figure 3, our SEM images confirm that these polymer structures had regular networks without irregular cross-linked networks. This experimental result is quite noticeable in that the height and density of the pPPy nanofibers and nanoparticles were significantly increased by the novel APPJ technique at room temperature. Furthermore, the proposed method also shows that the deposition rate was significantly increased by approximately 0.93 µm·min^−1^ under the room-temperature process. In other words, the proposed method indicates that the nano-size polymer can be grown rapidly during plasma polymerization without the thermal solution process.

**Figure 4:**
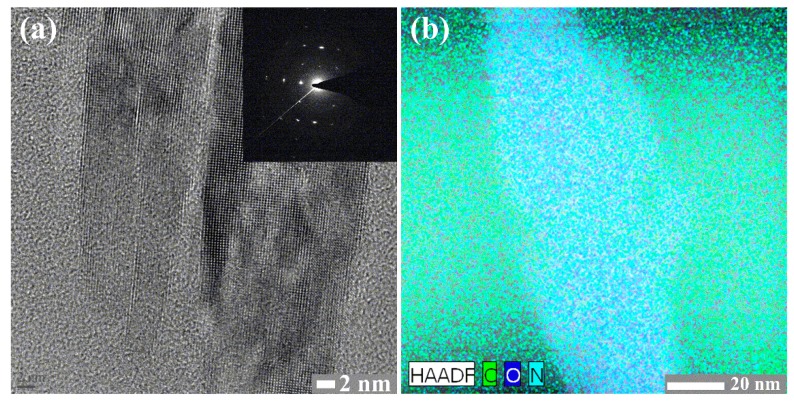
(**a**) Transmission electron microscopy (TEM) images of pPPy nanoparticles prepared via proposed APPJs, whose insulating substrate holder is placed inside the glass tube. High-resolution TEM images of single-crystalline pPPy nanoparticles; insets in (**a**) represent the selected area electron diffraction (SAED) pattern of pPPy nanoparticles; (**b**) High-angle annular dark-field scanning transmission electron microscopy (HAADF-STEM) and energy dispersive X-ray spectroscopy (EDS) elemental mapping images of C, O, and N. Scale bar = 2 nm (left) and 20 nm (right).

Figure 4a shows the TEM images of the pPPy nanoparticles grown in the inside case of Figure 1a. The pPPy nanoparticles, with a diameter range of 5–25 nm, were clearly observed. The selected area electron diffraction pattern of pPPy nanoparticles (Figure 4a, inset) reveals the clear diffraction spot indicating the single-crystalline structure. The characteristics of single-crystalline pPPy nanoparticles are attributed to regular-uniform and upright networks, as shown in Figure 3. The prepared nanostructures of pPPy visualized by energy dispersive X-ray spectroscopy (EDS) elemental mapping and high-angle annular dark-field scanning transmission electron microscopy (HAADF-STEM) are shown in Figure 4b. EDS and elemental mapping results reveal that the pPPy nanoparticles were exclusively composed of C, O, and N. Accordingly, the proposed device of APPJs can provide versatile advantages to the synthesis of polymer nanoparticle structures: (i) the plasma volume and intensity is increased due to the effect of the proposed glass tube and insulating substrate holder under ambient air (‘inside case’ of Figure 1a), resulting in higher plasma energies required for the synthesis of high-density polymer nanoparticles with single-crystalline characteristics; (ii) the polymer particles are changed to nanometer sizes due to the increased nucleation of the monomer caused by the intense plasma produced; and (iii) there is no thermal damage to the plastic substrate from the atmospheric pressure plasma polymerization due to its low temperature ionized discharge.

**Figure 5:**
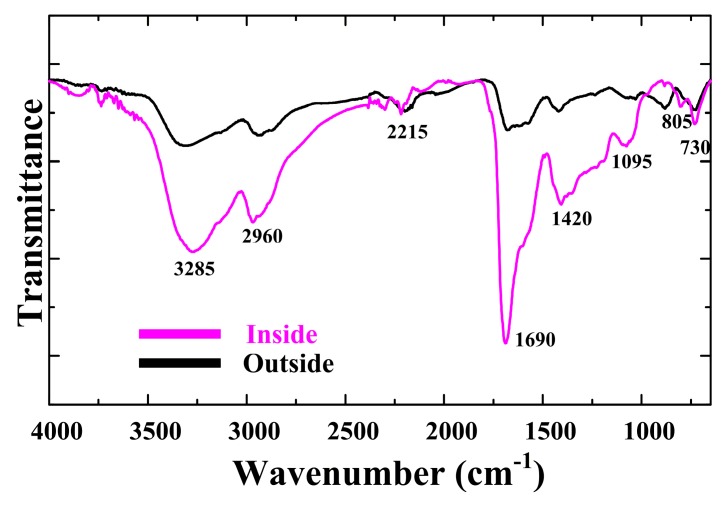
Changes in Fourier transform infrared spectroscopy (FTIR) spectra of pPPy nanofibers and nanoparticles thin film prepared using the proposed APPJs after a deposition of 60 min on plastic substrates in the outside and inside cases of Figure 1a, respectively.

FT-IR and XPS were used to determine the chemical changes introduced by the plasma. Figure 5 shows the FT-IR spectrum of the pPPy nanofibers and nanoparticle thin films on the plastic substrates after a deposition time of 60 min. In the inside case, the characteristic peaks were observed in the broad peak at 3285 cm^−1^ (N–H stretching with hydrogen bonded secondary amino groups) and 2960 cm^−1^ (aliphatic C–H stretching absorption). A peak belonging to the =C–H aliphatic vibration was located at 2215 cm^−1^. These peaks indicate that some of the pyrrole rings in the polymer were fragmented [37,38]. In the FT-IR spectra of pPPy, the different absorptions corresponded to alkenes, which resulted from broken rings and appeared as peaks 805 cm^−1^ and 730 cm^−1^, and the C=C double bond was found in the sharp peak at 1690 cm^−1^. The presence of these peak regions in pPPy films implies that the performance in electrical conductivity will be improved by doping carrier electrons [38,39,40].

**Figure 6:**
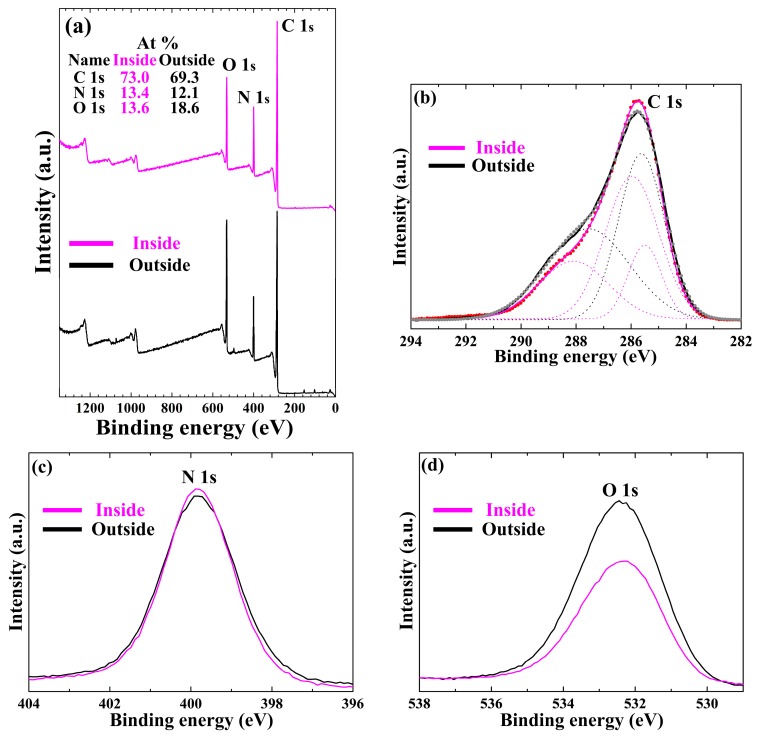
Differences in the inside and outside cases of pPPy nanofibers and nanoparticle thin film prepared using proposed APPJs after a deposition of 60 min on glass substrates. (**a**) X-ray photoelectron spectroscopy (XPS) survey spectra and detailed (**b**) C 1s (high resolution); (**c**) N 1s, and (**d**) O 1s spectra. Insets in (**a**) represent the atom percent in pPPy film. The XPS data is based in 1s orbitals.

Figure 6 contains XPS spectra and elemental composition (Figure 6a, insets) of the atomic distribution in the pPPy nanofibers and nanoparticle thin film on the glass substrates after a deposition of 60 min in outside and inside cases. As shown in Figure 6a, in the XPS survey spectrum, signals corresponding to C 1s (285.8 eV), N 1s (399.8 eV), and O 1s (532.4 eV) electronic orbitals can be observed. These results suggest that there are C, N, and O atoms in the pPPy thin film; the C and N atoms belong to the pyrrole structure, but the O atoms could have originated in the oxidation of the pPPy from ambient air. However, the additional weak emission lines at the binding energies of Zn (1072 eV), Na (500 eV), and Si (100–200 eV) can be only observed in the survey spectra of the outside case, which would be presumably due to the easy contamination from the ambient exposure and chamber. To obtain further insight into the chemical polar functional groups present on the surface of the pPPy thin films, curve fitting of the high-resolution C 1s peaks can be performed. Note that chemical assignments for deconvoluted peaks are based on the binding energies reported in the literature; 285.5 eV (C–C, C–H), 286.2 eV (C–N, C≡N), and 288.1 eV (O=C–N) [23,41,42]. As shown in Figure 6b, the 288.1 eV peak corresponding to carbons with oxidation from ambient air, such as O=C–N, was remarkably decreased in the inside case compared to the outside case, which showed more hydrophobic characteristics [43]. In addition, in the inside case, the C 1s, N 1s, and O 1s peaks had an atom percent of 73.0%, 13.4%, and 13.6%, respectively. Whereas, in the outside case, the C 1s, N 1s, and O 1s had an atom percent of 69.3%, 12.1%, and 18.6%, respectively. The C 1s and N 1s in the inside case were observed to have increased due to the presence of the proposed glass tube and insulating substrate holder. This indicates that the fragmented pyrrole rings in the inside case increased compared with those of the outside case. However, the O 1s in the inside case was observed to have decreased significantly, implying that the proposed glass tube and cylindrical insulating substrate holder contributed to minimizing the oxidation of pPPy from ambient air and therefore increasing the plasma energy in the nucleation region.

**Figure 7:**
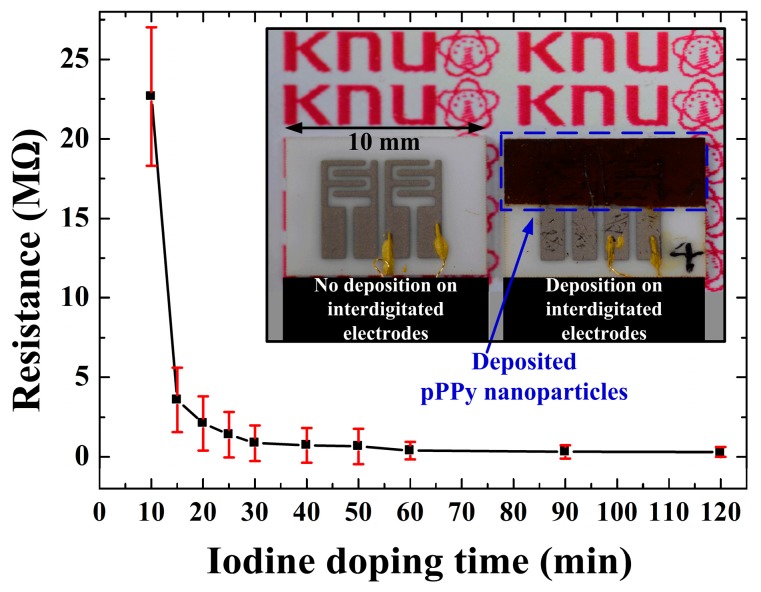
Changes in the resistance of pPPy nanofibers and nanoparticle thin films on substrates of interdigitated gas sensor electrodes under various iodine exposure (doping) times prepared using proposed APPJs with the insulating substrate holder placed inside the glass tube.

Figure 7 shows the resistance (*R*) of the pPPy nanofibers with nanoparticle thin films on the substrates of interdigitated gas sensor electrodes under various iodine exposure (doping) times under the same sheet thickness and area. The doping with iodine had the objective to introduce charge carriers into the pPPy structures for enhancing its electrical conductive characteristics. The resistance of the pPPy thin film was over 9 × 10^7^ Ω without iodine doping. As the doping time was increased, the corresponding resistance decreased sharply over a very short period, namely, 15 min, and was also saturated to be about 3 × 10^5^ Ω after 60 min. The as-synthesized pPPy is not electrically conductive, but its conductivity can be induced through an iodine doping process. Moreover, the resistance of the pPPy nanofibers and nanoparticle thin films prepared using the proposed APPJs can be easily controlled by simply doping with iodine under various doping times, which allows for their application in electrochemical toxic gas sensors due to their single-crystalline and porous nature. With this method, a detailed parametric study depending on humidity and temperature using various toxic gases will be carried out in the near future to measure the gas-sensing characteristics with electric conductivity of the nano-sized single-crystalline plasma polymers.

## 4. Conclusions

We demonstrated that the single-crystalline high-density pPPy nanoparticle structures were successfully synthesized on the glass, plastic and interdigitated gas sensor electrode substrates using novel APPJs at room temperature. As a result, the proposed APPJ device can obtain the high-density and ultra-fast pPPy thin films with single-crystalline nanoparticles for various gas sensor applications. Furthermore, we also expect that the new pPPy nanoparticles with the single-crystalline property grown under a low-temperature (30 °C) process can provide a versatile advantage for gas sensors, molecular electronics, future display technologies, optoelectronics, and bio-nanotechnology.
